# Evolutionary and plastic responses to climate change in terrestrial plant populations

**DOI:** 10.1111/eva.12112

**Published:** 2013-10-14

**Authors:** Steven J Franks, Jennifer J Weber, Sally N Aitken

**Affiliations:** 1 Department of Biological Sciences, Fordham UniversityBronx, NY, USA; 2 Department of Forest and Conservation Sciences, University of British ColumbiaVancouver, BC, Canada

**Keywords:** adaptive evolution, ecological genetics, global change, phenotypic plasticity

## Abstract

As climate change progresses, we are observing widespread changes in phenotypes in many plant populations. Whether these phenotypic changes are directly caused by climate change, and whether they result from phenotypic plasticity or evolution, are active areas of investigation. Here, we review terrestrial plant studies addressing these questions. Plastic and evolutionary responses to climate change are clearly occurring. Of the 38 studies that met our criteria for inclusion, all found plastic or evolutionary responses, with 26 studies showing both. These responses, however, may be insufficient to keep pace with climate change, as indicated by eight of 12 studies that examined this directly. There is also mixed evidence for whether evolutionary responses are adaptive, and whether they are directly caused by contemporary climatic changes. We discuss factors that will likely influence the extent of plastic and evolutionary responses, including patterns of environmental changes, species’ life history characteristics including generation time and breeding system, and degree and direction of gene flow. Future studies with standardized methodologies, especially those that use direct approaches assessing responses to climate change over time, and sharing of data through public databases, will facilitate better predictions of the capacity for plant populations to respond to rapid climate change.

## Introduction

There is now overwhelming evidence that climatic conditions are changing rapidly (IPCC 2007) and that plant populations are responding to these changes (Peñuelas and Filella 2001; Parmesan and Yohe 2003). For example, comparisons of contemporary flowering times of 43 plant species in Concord, Massachusetts to those recorded by Henry David Thoreau in the mid-nineteenth century showed an advance in flowering time of an average of 7 days as this location warmed by 2.4°C during this time period (Miller-Rushing and Primack 2008).

As the climate changes, plant populations may no longer be optimally adapted to new conditions (Anderson et al. 2012b; Shaw and Etterson 2012). Migration could allow some populations to track suitable conditions and thus maintain their adaptive optimum. However, this option may be limited, particularly in highly fragmented landscapes (Jump and Peñuelas 2005) and for species with long generation lengths (Aitken et al. 2008). Furthermore, migration may allow a population to stay within its climatic envelope but cause it to be out of synch with other environmental factors such as photoperiod (Bradshaw and Holzapfel 2008; McNamara et al. 2011) or key mutualists such as pollinators (Memmott et al. 2007; Hegland et al. 2009). To avoid extinction, many plant populations may need to respond to climate change through phenotypic plasticity or adaptive evolution (Holt 1990; Hoffmann and Sgro 2011).

The major questions are therefore: (i) Will plant populations be able to respond to contemporary climate change, and will these responses be fast enough to prevent extinction? (ii) To what extent do these responses reflect phenotypic plasticity versus adaptive evolution? and (iii) What factors influence whether the responses are adaptive or plastic? Addressing these questions is crucial for understanding and predicting how plant populations will respond to ongoing changes in climate, key knowledge for conserving both species diversity and evolutionary potential.

The main goal of this article is to review the evidence for plastic and evolutionary responses of plant populations to changes in climatic conditions. We define phenotypic plasticity as the ability of a genotype to express a different phenotype under different environmental conditions, and evolution as a shift in allele frequencies leading to a change in phenotype in a population (Conner and Hartl 2004). In practice, there are a number of different ways in which evolutionary and plastic responses to climate change can be detected (Box 1). We define adaptive evolution as an evolutionary change that leads to increased average fitness of individuals in a population in a given environment, with an evolutionary change not assumed to be adaptive without further evidence (Conner and Hartl 2004). We limit the scope of our review to terrestrial plants, which have long been subjects of studies on both adaptive evolution and plasticity [for reviews on other taxa, see in this issue: (Boutin and Lane 2014; Charmantier and Gienapp 2014; Reusch 2014; Schilthuizen and Kellermann 2014; Urban and Phillips 2014)]. Plants are highly amenable to these studies, because they can be transplanted, grown in common gardens, stored as seeds, cloned, crossed experimentally, and followed individually over time.

**Box 1: Methods commonly used for studying plastic and evolutionary responses to climate change**

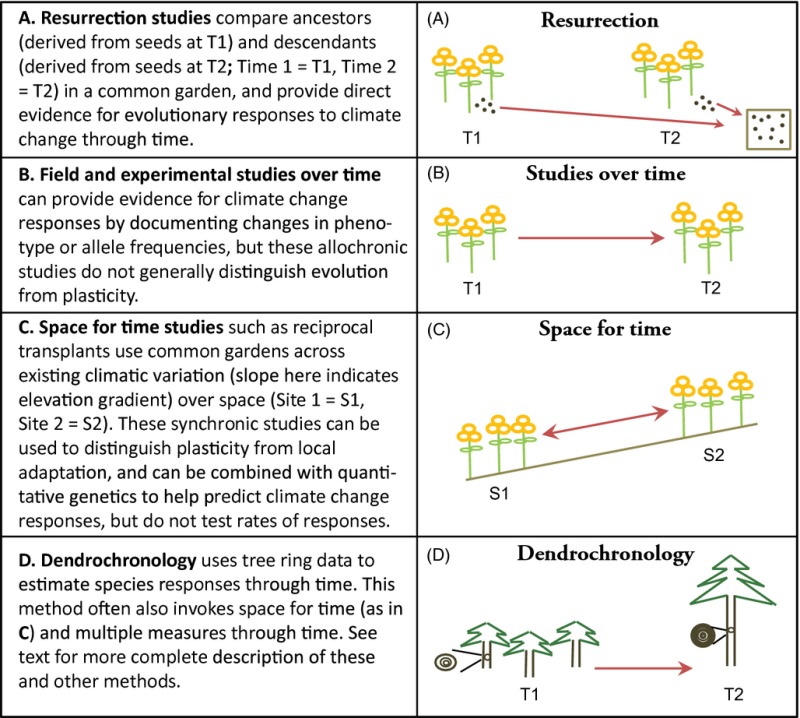

A change in phenotype over time as climatic conditions change could be the result of either evolution or plasticity, and neither should be assumed without further evidence (Merilä and Hendry 2014). While phenotypic shifts over time have been observed in a large number of species (Peñuelas and Filella 2001; Parmesan and Yohe 2003), it is not possible without additional information to determine whether these responses are due to plasticity or to evolution. Plasticity and adaptive evolution are not mutually exclusive (Nicotra et al. 2010). Some traits or populations may respond through plasticity, others through evolution, and others through some combination of the two. In addition, global change may induce the evolution of plasticity, with traits evolving to become more or less plastic as environmental conditions change. Evolutionary changes in plasticity can further complicate predictions of climate change responses. For example, one study showed that selection could cause the evolution of increased or decreased plasticity, depending on the environmental conditions (Springate et al. 2011). Both plastic and evolutionary responses to climate change can influence population persistence, and both may or may not be adaptive (Ghalambor et al. 2007). Furthermore, plasticity and adaptation may interact. Plasticity can hinder evolution by dampening the effects of selection, or promote evolution by allowing population persistence under changing conditions (Chevin and Lande 2010, 2011; Chevin et al. 2010, 2013; Crispo et al. 2010; Thibert-Plante and Hendry 2011; Kovach-Orr and Fussmann 2013). Thus, making precise quantitative predications about the rate and extent of plastic and evolutionary responses to climate changes in natural plant populations is complex.

In this article, we first provide an overview of the literature on local adaptation to climate in plants, because the degree of local adaptation in the past to variation in climatic conditions can be used to help predict the capacity for genetic responses to climatic change in the future. Next, we review general patterns in studies that have examined evolutionary and plastic responses to climate change, followed by case studies highlighting several key methods. Finally, we discuss factors influencing the type and rate of climate change response, and future directions in research.

## Local adaptation to climate in plants

To determine how plants will respond to changes in climate, it is useful to draw on the extensive body of work on local adaptation to variation in climatic conditions. A central goal in ecological genetics has been to determine to what extent different phenotypes in different environments result from local adaptation, phenotypic plasticity or some combination of the two (Conner and Hartl 2004; Ghalambor et al. 2007). If populations are locally adapted, then they may suffer a fitness decline as climatic conditions change because these populations may not be adapted to the new conditions. Thus, even if a species occurs across a range of climatic conditions and the new climate for a given population remains within the broader climate envelope of that species, locally adapted populations could suffer fitness declines with climate change. Such a scenario has been shown in two recent studies (Etterson 2004; Kim and Donohue 2013). However, local adaptation could also provide evidence of the capacity of populations to adapt to new conditions. Finding local adaptation also suggests that alleles for adaptation to new conditions may exist elsewhere in a species range. In any case, the extent of local adaptation to climatic conditions is highly relevant for predicting future responses to climate change.

How widespread is local adaptation to climate in plants? The definitive test for local adaptation is the reciprocal transplant experiment. Two meta-analyses of reciprocal transplant studies, one including 35 studies of only plants (Leimu and Fischer 2008), and one including 50 plant and 24 nonplant studies (Hereford 2009) have both found that local adaptation is common but not universal, with 71% of studies in both analyses showing some evidence of local adaptation. Leimu and Fischer (2008) found that small populations were much less likely to show local adaptation than large populations, as would be predicted by theory (Willi et al. 2006) because drift increases in strength relative to selection with decreasing population size. Local adaptation in plants is often attributed specifically to variation in climate between populations or test environments. Of the 50 plant studies included in Hereford's (2009) analysis, 26 specified focal or suspected environmental drivers of divergent selection between sites, and 22 of these (85%) included climatic variables or closely related surrogates such as elevation or soil moisture. Many tree species have shown strong evidence of local adaptation to climate in provenance experiments, which are common garden experiments testing the effects of the place of seed origin (provenance) on tree survival, growth rates, and other phenotypic traits. In a meta-analysis of provenance experiments for 59 tree species and 19 phenotypic traits, some of which are partial reciprocal transplants with home sites for a subset of provenances, 90% of analyses found significant population differentiation for phenotypic traits, and in most cases, this differentiation was correlated with and attributed to variation in climate (Alberto et al. 2013).

Reciprocal transplant experiments and other common gardens assessed at a single point in time (synchronic studies) substitute space for time when used as climate change experiments and do not directly assess the capacity for and maximum rate of rapid local adaptation to contemporary climate change (Box 1). However, they can indicate whether or not plant populations have locally adapted to climate in the past (e.g., during recolonization since the last glacial maximum), and they can provide valuable information about phenotypic plasticity if individuals from the same genetic group (populations, families or clones) are planted experimentally in multiple environments and data are collected to determine whether traits of cloned genotypes or related individuals vary across environmental conditions. These experiments can help to identify the specific components of climate (temperature and moisture variables) that are likely drivers of divergent selection or plasticity, which can then be further investigated with manipulative experiments. Transplant experiments can also facilitate the identification of genetic markers associated with local adaptation to climate that would be appropriate for tracking adaptation to climate change allochronically (over time) (Wilczek et al. 2010; Fournier-Level et al. 2011). While reciprocal transplant studies primarily provide opportunities to study phenotypic plasticity in a synchronic context, they can also provide a setting for monitoring phenotypic plasticity in long-lived individuals over time. For example, dendrochronology studies (Savva et al. 2008; McLane et al. 2011) can be used to assess variation in tree rings in common garden experiments (Box 1), although ontogenetic effects of aging need to be separated from effects of temporal fluctuations in climate.

These previous studies indicate that many populations are locally adapted to climatic conditions that vary over space, and that evolution in response to climatic variation has occurred in the past. We also know that phenotypic plasticity is extensive, and that plants can exhibit plastic responses to climatic variation (Nicotra et al. 2010). We now assess the current literature on plant plastic and evolutionary responses to ongoing rapid climate change and attempts to predict future responses to changes in climatic conditions.

## Literature review

We searched the literature using Web of Science (Thomson Reuters, New York, NY, USA) and Google Scholar (Google, Mountain View, CA, USA) with the search terms ‘plasticity,’ ‘evolution,’ ‘adaptation,’ ‘selection,’ ‘climate change,’ ‘global change’ and ‘plant’. We screened the resulting publications, excluding any that did not fit the criteria outlined by Merilä and Hendry (2014). Studies in which the authors were not specifically aiming to examine evolutionary or plastic responses to climate change were not included, even if they fit the Merilä and Hendry criteria. We included other studies we knew examined evolutionary or plastic responses to climate change in plant populations, even if these did not show up in our initial search.

While any study examining the extent of local adaptation to climatic conditions or determining selection on or genetic variation for traits related to climate adaptation could potentially be used to predict potential evolutionary responses to climate change, we only included in this review those studies that could test whether evolutionary responses to contemporary climate change had actually occurred. For example, any study involving a reciprocal transplant among populations experiencing different climatic conditions, for example, (Clausen et al. 1940) could possibly be used, with the ‘space for time substitution approach,’ to infer potential future responses to climate changes. Such studies were not included in our quantitative review if the authors did not intend to evaluate evolutionary or plastic responses to climate change, and it was beyond the scope of this review to include all studies showing any link between traits and climate. We included both field studies and experiments conducted under simulated climate change conditions (such as in controlled environment chambers). Studies examining only phenotypic variation or only genotypic changes were also included. There are likely some studies that would fit our criteria that were not picked up in our search, and we did not attempt to be comprehensive. However, we expect that our strategy allowed us to review a representative sample of studies directly examining evolutionary or plastic responses to climate change in plant populations.

We found 38 studies examining plastic or evolutionary responses to climate change in plant populations that met our criteria (Table 1). Though modest, this is a relatively large number of studies aimed at investigating plastic and genetic responses to climate change in comparison with those included in reviews for other groups in this issue, for example, 19 studies for mammals (Boutin and Lane 2014) and 14 studies for birds (Charmantier and Gienapp 2014). While this relatively small number of studies limits our ability to draw firm conclusions, we can begin to detect some patterns and trends. The number of studies in this area is growing quickly. The earliest studies were published in 1991, and 31 of the 38 studies have been published since 2007 (Table 1). The number of studies on this topic will likely continue to increase rapidly (Parmesan 2006; Merilä 2012).

**Table 1:** Summary of studies of terrestrial plants designed to examine plastic and/or genetic responses of traits driven by climate change

Family	Species	Trait type	Genetic	Plastic	Adapt	Cause	Time	References
A. Studies showing direct evidence for genetic and/or plastic changes due to climate change								
Brassicaceae	*Brassica rapa*	PH, PY	Y(2,3)	Y(4)/N(2,3)	Y(1,2)	Y(2,3)	RS	Franks et al. (2007), Franks and Weis (2008), Franks (2011)
Lamiaceae	*Thymus vulgaris*	PF	Y(6)	.	Y(2)	Y(1)	FD	Thompson et al. (2007, 2013)
Poaceae	*Andropogon gerardii*	PY, GR	Y(2,3,6)	Y(2,3,4)	Y(3)*	Y(3)	EX	Avolio et al. (2013), Avolio and Smith (2013)
Poaceae	*Triticum dicoccoides* and *Hordeum spontaneum*	PH, AF	Y(2,6)	.	.	Y(2)	RS	Nevo et al. (2012)
Polygonaceae	*Polygonum cespitosum*	PY, GR	Y(2,3)	Y(2,3)	Y(1,2)*	Y(2,3)	RS	Sultan et al. (2013)
B. Studies showing strongly suggestive evidence for genetic and/or plastic changes due to climate change								
Betulaceae	*Betula pubescens* and *Betula pendula*	PH	Y(2,3)	Y(2)	Y(2)†	Y(1)	EX	Billington and Pelham (1991)
Brassicaceae	*Arabidopsis thaliana*	PH	Y(2,4)	Y(2,3)	N(2)	Y(2,3)	EX	Springate et al. (2011)
Brassicaceae	*Boechera stricta*	PH	Y(2,3)	Y(2,4)	Y(1,2)†	Y(2)	EX, FD	Anderson et al. (2012a)
Brassicaceae	*Brassica juncea*	PH, GR	Y(2,3,4)	Y(2,3)	N(1,2)	Y(3)	EX	Potvin and Tousignant (1996)
Brassicaceae	*Erysimum capitatum*	GR	Y(2,5)	Y(2,4)	Y(1)†	Y(1)	EX	Kim and Donohue (2011)
Caryophyllaceae	*Colobanthus quitensis*	PY, GR	Y(2,4)	Y(2,3)	Y(1)*	.	EX	Molina Montenegro et al. (2012)
Fagaceae	*Fagus sylvatica* and *Quercus petraea*	PH	Y/N(2,5)	Y(2,5)	.	Y(1)	EX	Vitasse et al. (2010)
Fagaceae	*Quercus suber*	PY, GR	Y(2,5)	Y(2,4)	Y(1)	Y(2)	EX	Ramírez-Valiente et al. (2010)
Myrtaceae	*Eucalyptus globulus*	PY	Y(2,3,5)	.	Y(4)	Y(2)	EX	Dutkowski and Potts (2012)
Poaceae	*Festuca lenensis*	GR	Y(2,5)	Y(2,3,4)	Y(1) †	Y(2)	EX	Liancourt et al. (2012)
Rhizophoraceae	*Rhizophora mangle L*.	PY, GR	.	Y(3,5)	.	Y(3)	EX	Ellison and Farnsworth (1997)
Betulaceae	*Betula pendula*	AF	Y(6)	.	.	Y(2)	DO	Kelly et al. (2003)
Fagaceae	*Fagus sylvatica*	AF	Y(5,6)	.	.	Y(2)	DO	Jump et al. (2006)
Pinaceae	*Pinus banksiana*	GR	Y(2)	Y(2,4)	.	Y(2)	DO	Savva et al. (2008)
Pinaceae	*Pinus contorta*	GR	Y(2,5)	Y(2,5)‡	.	Y(2)	DO, FD	McLane et al. (2011)
Pinaceae	*Pseudotsuga menziesii*	GR	Y(2,3)	Y(2,4,5)	Y(2)	Y(2)	DO, MD	Martinez-Meier et al. (2009)
Mutiple	27 different sp.	AF	Y(5,6)	.	.	Y(1)	MD	Alsos et al. (2012)
Pinaceae	*Abies sachalinensis*	GR	Y(2,5)	Y(2,5)	Y(1) †	Y(1)	MD	Ishizuka and Goto (2012)
Pinaceae	*Pinus contorta*	GR	Y(2,3)	Y(2,5)*	.	Y(1)	MD	Wang et al. (2010)
Pinaceae	*Pinus sylvestris*	PH	Y(2,3,5)	Y(2)*	Y(2) †	Y(2)	MD	Savolainen et al. (2004)
Pinaceae	*Pinus sylvestris*	PH, AF	Y(2,5,6)	Y(2,4)	Y(1,2)	Y(1)	MD	Savolainen et al. (2007, 2011)
Asteraceae	*Artemisia californica*	PH, PY, GR	Y(2,3,5)	Y(2,3,4)	Y(1,2) †	Y(2,3)	.	Pratt and Mooney (2013)
Brassicaceae	*Brassica rapa*	PH, GR	Y(2)	Y(2)	Y(1)	Y(3)	.	Lau and Lennon (2012)
Fabaceae	*Chamaecrista fasciculata*	PH, GR	Y(2,3,5)	Y(2,4)	Y(1,2)†	Y(1)	.	Etterson and Shaw (2001), Etterson (2004)
Phrymaceae	*Mimulus laciniatus*	GR	Y(2,5)	.	Y/N(2)	Y(1)	.	Sexton et al. (2009)
Pinaceae	*Picea sitchensis*	PH, GR	Y(2,4,5)	Y(2,3,4)	Y(1)	Y(2)	.	Mimura and Aitken (2010)
Pinaceae	*Picea sitchensis* × *P. glauca*	AF	Y(3,5,6)	.	.	.	.	Hamilton et al. (2013)
Pinaceae	*Pinus pinaster*	PY	Y(2,5)	Y(2,4,5)	.	Y(2)	.	Corcuera et al. (2011)
Pinaceae	*Pinus sylvestris*	GR	Y(2,3)	Y(2,3)	.	Y(1)	.	Richter et al. (2012)
Poaceae	*Festuca eskia*	GR, AF	Y(2,5,6)	Y(2,4)	Y(1)*	Y(2)	.	Gonzalo-Turpin and Hazard (2009)
Salicaceae	*Populus balsamifera L*.	PH, PY	Y(3,5)	.	Y(4)	Y(2)	.	Keller et al. (2011)
Multiple	4 different sp.	PY, GR	.	Y(3)	.	.	.	He et al. (2007)
Mutiple	57 different sp.	PH	.	Y(3)	.	Y(1)	.	Cleland et al. (2012)

The 38 studies include five providing strong evidence (A) and 33 providing strongly suggestive evidence (B), based on the criteria of Merilä and Hendry (2014). Shown are Family and Species (genus and species) of the focal plant, and Trait type (type of trait that showed a response to climate change): PH – phenology, PY – physiology, PF – frequency of genetically controlled phenotype, GR – observed responses in some measure of growth (e.g., biomass, stem count, leaf width, reproductive output), AF – allele frequencies or genetic markers. Also given are information on Genetic (evolutionary) and Plastic responses, and whether these responses are Adaptive and Caused by climate change. For genetic and plastic responses, ‘Y’ indicates that evidence was found; ‘N’ indicates that evidence was not found; ‘.’ indicates that it was not investigated. For Adaptive, ‘Y’ indicates that responses increased fitness or were predicted to increase fitness in new climatic conditions; ‘N’ indicates maladaptive responses; ‘.’ indicates that adaptation was not investigates; ‘^†^’ notes that adaptation was found but was not predicted to be sufficient to keep up with climate change; * notes that adaptation was predicted to be sufficient to keep up with climate change. For Cause, ‘Y’ indicates that the response was directly caused by climate change; ‘.’ indicates that causality was not investigated. Numbers denote the method of investigation invoked. Genetic categories: 2 – Common garden studies, 3 – Comparison to model predictions, 4 – Experimental evolution, 5 – Space for time substitution, 6 – Molecular genetic approaches; Plastic categories: 2 – Common garden studies, 3 – Experimental studies, 4 – Fine-grained population responses, 5 – Individual plasticity in nature; Adapt categories: 1 – Reciprocal transplants, 2 – Phenotypic selection estimates, 3 – Genotypic selection estimates, 4 – *Q*_st_-*F*_st_ comparison; Cause categories: 1 – Common sense, 2 – Phenotype by environment interactions, 3 – Experimental selection/evolution. For full descriptions of all categories see Merilä and Hendry (2014). Time (type of approach using a time component in data collection): RS – resurrection study, EX – field or greenhouse experiment through time, FD – field observations through time, MD – modeled through time, DO – dendrochronology (tree ring data over time), ‘.’ indicates no temporal component.

* Adaptation predicted to be sufficient to keep up with climate change.

† Adaptation not predicted to be sufficient to keep up with climate change.

‡ Used a modeling approach to test for plasticity.

The studies included diverse taxa but were not a taxonomically representative sample of terrestrial plant species. There were 11 studies on Pinaceae, six on Brassicaceae, 11 other families represented in three or fewer studies, and three studies performed on multiple families (Table 1). Life form was also varied, with 20 studies of trees, 14 studies of herbaceous plants, and four studies of grasses (all Poaceae).

A variety of traits were examined in these studies. Fifteen examined phenology, which is known to be strongly influenced by climate change (Parmesan and Yohe 2003), particularly for phenological traits that are triggered by chilling or degree days. Twenty-one examined growth (e.g., biomass, leaf width, stem count); 11 measured physiological traits (e.g., drought tolerance, photosynthetic rate); seven evaluated changes in allele frequencies via genes or genetic markers; and one study assessed frequency of a chemical phenotype. Many of these studies examined more than one category of trait (Table 1).

The studies also took a variety of scientific approaches (Box 1) that were outlined by Merilä and Hendry 2014. There were 35 studies examining genetic and evolutionary change, and of these 29 studies used common gardens, 15 used comparisons to predict quantitative genetics models (Lande 1979; Lande and Arnold 1983), four used experimental evolution, 19 used space for time substitution (such as reciprocal transplants or sites across an altitudinal gradient), seven used molecular genetic approaches, and two used a phenotypic marker that could be directly linked to an underlying gene (Table 1). There were 29 studies examining plastic responses, and of these, 26 used common gardens, 13 used field or greenhouse experiments, 14 used fine-grained population responses, and seven used individual plasticity in nature. Most studies combined multiple approaches.

**Figure 1:**
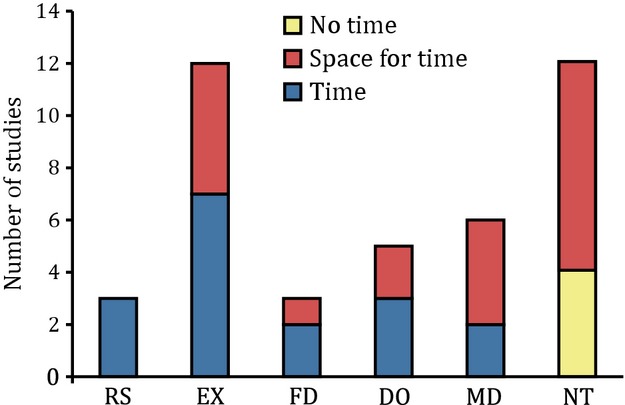
Analysis of time component in studies examining evolutionary and plastic responses to climate change in terrestrial plants. Shown are the number of studies taking the approaches of resurrection studies (RS), field or greenhouse experiments (EX), measurements in natural populations (FD), dendrochronology studies (DO), modeling (MD), and those that did not have any synchronic temporal component (NT). Studies that also used space for time substitution (allochronic) along with other approaches are shown in red (top portion of bars). Note that several studies took multiple approaches, and each time an approach was taken it is shown here, so the total tally in the figure exceeds the total number of studies in the review (*n* = 3 studies used multiple synchronic time components).

The most direct evidence for evolutionary or plastic responses to climate change comes from data collected over time. Many of the experimental plant studies we have included had a temporal component to elucidate genetic and plastic responses to climate change (Fig. 1). There were 26 (72%) studies that had some temporal component to their analyses (Fig. 1). Seventeen of these (65%) directly measured trait changes over time, with three studies using the resurrection approach (Davis et al. 2005; Franks et al. 2008) of comparing ancestors and descendants under common conditions, 11 using controlled field or greenhouse or growth chamber experiments, one directly observing changes in phenotypes in field studies, one combining field and controlled experiments, and one combining field observations and dendrochronology (tree ring analysis; Table 1). Ten studies used dendrochronology and/or modeling approaches to understand genetic and plastic responses to climate change.

For those studies that directly measured changes in phenotype or genotype over time, the duration of the studies varied widely. For example, nine of 10 field or greenhouse experiments (90%) lasted 3 years or less. Two experimental evolution studies were carried out for five and seven generations. Studies that were considered to provide the most direct evidence for genetic and plastic responses to climate change included common gardens followed through time or direct observation of a change in allele frequency through time, (see Merilä and Hendry (2014) for more detailed description of categories; Table 1a). Five studies provided this direct evidence, with traits measured in populations over much longer time periods than in other experimental studies included here, lasting between 7 and 36 years. Notably, dendrochronology studies can also span long periods, using the records of growth over time laid down in tree rings in long-lived woody perennials.

A major objective of this review was to determine how frequently evolutionary or plastic responses to climate change occur. Evolution was examined in 35 of the studies, and at least some evidence for evolutionary response to climate change was found in all 35 of these (100%; Table 1). Plasticity was examined in 29 of the studies, and all 29 of these (100%) found some evidence for plastic responses to climate change (Table 1). Thus, in almost every case where evolution or plasticity was investigated, it was found.

These results indicate that both plastic and evolutionary responses to climate change can occur. However, there are important caveats. First, any study showing an evolutionary or plastic response in one trait was scored as finding that response, even if a response was not detected in another trait in that study. This could potentially overestimate positive responses. However, in the majority of studies that measured multiple traits, most traits showed a genetic or plastic response (Gonzalo-Turpin and Hazard 2009; Keller et al. 2011). Second, there could be publication bias (the file drawer problem), also causing an overestimate of positive responses (Møller and Jennions 2001). Third, even if evolutionary or plastic responses to climate change occurs, this does not necessarily mean that the responses would be sufficient to keep pace with the current rate of climate change. In fact, we found 12 studies that investigated this, and eight concluded that the responses expected would not be sufficient given predicted rates of climatic changes (Billington and Pelham 1991; Etterson 2004; Savolainen et al. 2004; Jump et al. 2006; Anderson et al. 2012a; Liancourt et al. 2012; Kim and Donohue 2013; Pratt and Mooney 2013), while four studies predicted that responses can potentially keep up with climate change (Gonzalo-Turpin and Hazard 2009; Molina Montenegro et al. 2012; Avolio et al. 2013; Sultan et al. 2013). Thus, the high apparent frequency of plastic or evolutionary responses should not be taken as a clear indication that populations can generally keep pace with climate change.

In addition to assessing how frequently evolutionary and plastic responses occurred, we wanted to find out if these tended to be alternative or co-occurring strategies. We found 26 studies that examined both plasticity and evolution, and all 26 of these (100%) showed some evidence for both plastic and evolutionary responses (Table 1). The remaining 12 studies that showed only an evolutionary or a plastic response did not investigate the other type of response. Thus, there was no evidence that a plastic response precluded an evolutionary response, or *vice versa*. Plasticity and evolution occur simultaneously, and these are not alternative or mutually exclusive responses. Only two studies (Wang et al. 2010; Anderson et al. 2012a) attempted to partition the amount of phenotypic change due to evolution versus plasticity, and both showed contributions of both plasticity and evolution.

Merely finding an association between a phenotypic change and a climatic change does not necessarily show causality. In our survey, 35 of the 38 studies inferred causality between climate change and evolutionary or plastic responses. However, in 12 of these studies (34%), the inference was based on common sense alone. Thus, it is not always clear that changes in climate are actually the cause of observed plastic or evolutionary shifts in phenotype. Similarly, if an evolutionary change occurs, it is not necessarily the case that the change was adaptive. In our survey, evidence for adaptive evolution resulting in increased mean fitness in new or predicted climates was shown in 21 studies, and two studies found evidence for a lack of adaptive evolution. In at least one study (Sexton et al. 2011), adaptation was context-dependent. Adaptation was not investigated in the remaining 14 studies. A relatively high proportion of studies found evidence of evolutionary changes, but clear evidence that adaptive evolutionary shifts were directly caused by climate change was somewhat less common [but see: (Franks et al. 2007; Thompson et al. 2007; Franks 2011; Avolio et al. 2013; Avolio and Smith 2013; Sultan et al. 2013; Thompson et al. 2013)].

The relative amount of phenotypic variation explained by plasticity versus genetic variation depends on the climatic range of populations sampled and the environments they inhabit, as well as the climatic range of test site environments and details of the experimental designs. Most studies used reciprocal transplant studies or experimental environment common gardens to assess phenotypic plasticity and included genotypes from different populations, families, or lines, allowing for some qualitative comparisons of the relative effects of environment versus genotype on phenotypes. However, given the wide range of experimental approaches, it is difficult to develop a standard metric for quantitatively comparing the magnitude of plasticity versus genetic responses across studies.

Within experiments, traits vary considerably in the relative amounts of phenotypic variation explained by plasticity versus genetic group [population, family, or recombinant inbred line (RIL)]. For example, one study (Potvin and Tousignant 1996) subjected two populations of *Brassica juncea* to selection in environments simulating pre- and postglobal warming climates and assessed 14 traits after seven generations of selection. They found that phenotypic responses to the simulated warming in some traits were largely the result of plasticity, while other traits mainly varied genetically, and others showed similar levels of phenotypic variance resulting from genetic variation and plasticity. However, none of these responses were sufficient to adapt these populations to predicted future conditions. The authors attributed this lack of response to inbreeding depression.

This quantitative review highlights some of the major trends across studies in plastic and evolutionary responses to climate change. The studies included show that plastic and evolutionary responses are occurring and are being detected through a variety of methods. These methods and results are illustrated in the following case studies.

## Case studies

### Contrasting approaches to investigating evolution

We highlight here a few key studies that have advanced our understanding of evolutionary responses to climate change using a variety of approaches. They include studies using natural plant populations, constructed genetic lines, historical data, previously collected seeds, phenotypic selection analysis, and genetic markers and genomics. This set of approaches is applicable to investigating not just climate change responses but contemporary evolution in general.

While most studies of evolution in natural populations use wild individuals, one study (Anderson et al. 2012a) of evolutionary responses to climate change used RILs. Recombinant inbred lines have the advantage of producing a large amount of genetic and phenotypic diversity, facilitating the estimation of selection and of additive genetic variation. The authors planted RILs of the Arabidopsis relative *Boechera stricta* in the field and measured selection and quantitative genetic variation in these experimental populations. They then combined this quantitative genetic information with historical data on flowering time of this species in this region. The investigators found that flowering time in this species had advanced significantly, by 0.34 days per year on average, between 1973 and 2011. This response is consistent with shifts in phenology seen in a variety of life forms in response to global warming (Parmesan and Yohe 2003; Charmantier and Gienapp 2014; Schilthuizen and Kellermann 2014; Urban and Phillips 2014). Using quantitative genetic analyses, Anderson et al. (2012a) found that strong directional selection favored earlier flowering in contemporary environments. They estimated that 20% of the phenotypic change was due to evolution. They concluded that plasticity was important in flowering timing and its shift over recent decades.

**Figure 2:**
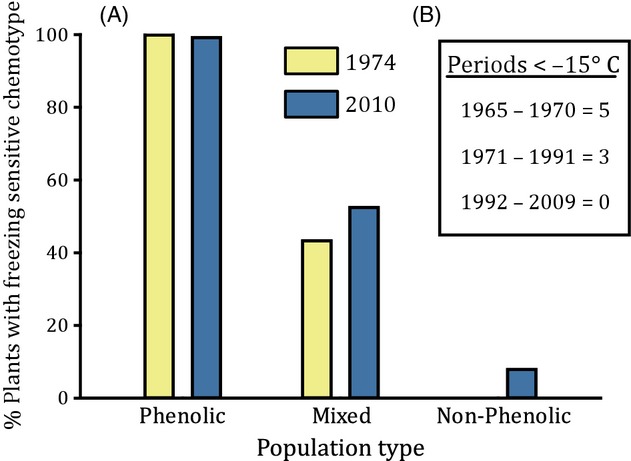
Changes over time in the frequency of freezing sensitive chemotypes of *Thymus vulgaris* (Lamiaceae) with climatic warming (Thompson et al. 2013). Populations along altitudinal transects in France were first surveyed in 1974 and again in 2010. The proportion of individuals with a freezing sensitive chemotype increased between 1974 (yellow bars, left) and 2010 (blue bars, right), especially in populations that were initially frost tolerant (nonphenolic) or mixed (Fig. 2A). This corresponds with a marked decrease in episodes of severe frost (temperatures < −15°C) between 1965 and 2010 (Fig. 2B). Because the chemotypes are entirely genetically determined (not plastic), this provides compelling evidence of evolutionary change with changing climatic conditions.

Another recent study (Thompson et al. 2013) also took advantage of an existing dataset from decades earlier, in this case, on chemotypes (chemical phenotypes) of the woody perennial wild thyme (*Thymus vulgaris*). The different chemotypes have differential sensitivity to cold, with the phenolic chemotypes more freezing sensitive and the nonphenolic chemotypes more freezing tolerant. The phenolic chemotype may be more resistant to herbivory (Linhart and Thompson 1995). Data from 1974 indicated variation among populations along altitudinal transects in the extent of phenolic and nonphenolic chemotypes, with areas experiencing colder temperatures generally made up of nonphenolic chemotypes, and warmer areas comprised of phenolic chemotypes (Fig. 2A). The previously colder locations saw a decrease between 1965 and 2009 in the frequency of very cold periods (<−15°C) (Fig. 2B), as well as an increase in the proportion of individuals with freezing sensitive chemotypes (Fig. 2A). This result indicates that warming temperatures have led to evolutionary shifts in the population frequencies of freezing sensitive chemotypes. Because these chemotypes are entirely genetically determined rather than plastic, this is clearly a case of an evolutionary response to changing climatic conditions.

Rather than using only pre-existing data, some studies have used previously collected seeds and compared ancestors and descendants under common conditions to directly assess evolutionary responses to climate change through the resurrection approach (Davis et al. 2005; Franks et al. 2007, 2008). The resurrection approach was recently used to examine evolutionary responses to climate change in wild wheat (*Triticum aestivum*) and barley (*Hordeum vulgare*) populations in Israel (Nevo et al. 2012). Plants grown from seeds collected in 1980 were compared with those grown from seeds collected in 2008. Flowering time was significantly earlier in both species in the 2008 samples than in the 1980 samples, by an average of 8.5 days in wheat and 11 days in barley. Although this study did not directly determine the cause of this phenological change, it is consistent with shifts in flowering time seen in many other species following global warming and increased drought (Parmesan and Yohe 2003). The Nevo et al. (2012) study also examined microsatellite (SSR) genetic markers and found substantial genetic divergence between the ancestral and descendant populations. These results confirm both phenotypic and genetic shifts following a change in climate.

To assess evolutionary responses in long-lived plants like forest trees, individuals do not necessarily need to be resurrected from the past because some are still standing. One study taking advantage of this fact used dendrochronology (tree ring) data to estimate ages of *Fagus sylvatica* individuals in Catalonia, Spain (Jump et al. 2006). They used amplified fragment length polymorphism scans to assess variation throughout the genome and to associate this genetic variation with climatic variation and changes over time. As temperatures increased in this area over the last half-century, the frequency of a genetic marker associated with variation in temperature declined by an average of 0.135/°C. While this result indicates an evolutionary response to selection, the authors caution that their data indicate that this response would likely not be sufficient to allow the populations to adapt to climate change throughout their range (Jump et al. 2006).

Similar concerns about the capacity of plant populations to adapt to changing climatic conditions were raised in a study of *Chamaecrista fasciculata* (Etterson and Shaw 2001). Reciprocal transplant experiments and quantitative genetic analyses were used to assess the ability of populations to respond to changes in climate. They found genetic correlations between traits opposed the direction of selection for the traits, indicating that evolutionary responses would likely be constrained by genetic architecture. Studying a single trait would not have revealed this constraint.

Based on these studies, it is clear that climate change is a potent agent of selection that can result in evolution on a short timescale. However, there are also substantial constraints. It remains unclear to what degree evolution will allow plant populations to keep pace with ongoing climate change.

### Separating plastic and adaptive responses to climate using long-term provenance trials

There is a wealth of data from long-term provenance (geographic or climatic seed source origin) reciprocal transplant trials for forest trees (Alberto et al. 2013). Although many of these were designed and established before climate change was recognized as a major global problem, these trials can be analyzed retrospectively with new approaches to understand the extent of local adaptation to climate by substituting climatic variation in space for time, and to identify phenotypes that are locally adapted and the climatic factors they are adapted to (Box 1). A space for time approach allows genotypes to be studied over a broader range of climatic conditions than short-term temporal studies sample (Teplitsky and Millien 2014), but observations at a single point in time do not permit the direct study of evolutionary responses as climate changes. Some provenance trials have been measured repeatedly over time and are planted on contrasting field sites, allowing for both of these approaches to be utilized.

The Illingworth lodgepole pine (*Pinus contorta*) provenance trial in British Columbia, Canada provides a useful example of this (Rehfeldt et al. 1999, 2001). Seeds were collected from 120 locations across the species range and planted in 60 field sites in 1974. Data on tree growth have been collected for over three decades. Although each provenance was only planted at a subset of sites, it has been possible to derive population reaction norms to site climatic variables (Rehfeldt et al. 1999, 2001; Wang et al. 2010). Using this experiment, researchers developed a ‘universal response function’ approach to describing phenotypic variation as a multidimensional function of provenance climate and site climate (Wang et al. 2010). With this function, they were able to estimate the proportion of phenotypic variation due to site climatic conditions (phenotypic plasticity) versus provenance climatic conditions (genetic variation reflecting adaptation). Similar to the results of Anderson et al. (2012a), genetic variation explained about 20% of the phenotypic variation, with phenotypic plasticity explaining the majority of variation in response to test site temperature.

While provenance trials are usually synchronic, substituting space-for-time when used to examine responses to climate change, dendrochronological analyses of the annual growth rings of trees allow for the analysis of phenotypic plasticity in response to temporal climatic variation (Box 1). The universal response function approach was applied to annual growth ring data taken from wood cores sampled from a subset of materials in the Illingworth trial for the analysis of climatic sensitivity (intraindividual plasticity) in response to annual and seasonal variation in climate (McLane et al. 2011). Average tree ring widths over the 23-year chronology showed strong interactions between mean temperature of population origins and mean temperature of test sites, reflecting local adaptation, and strong effects of site temperature and moisture, reflecting phenotypic plasticity. Annual variation in ring width (climatic sensitivity) showed significant but somewhat weaker effects of site climate and provenance climate than average ring width.

### Phenology

Phenology of many traits in plants outside of the tropics is a function of seasonal thermal or photoperiodic cues, some of which can be modified by other environmental stresses such as drought. Seven of the studies we compiled examined phenotypic plasticity in phenological traits including the timing of flowering, leafing out or bud break in spring, and bud set or leaf senescence in fall, and all found evidence of phenotypic plasticity (Table 1). Spring phenological traits, such as timing of bud break, first leaf emergence, and spring flowering of temperate and boreal perennials and winter annuals, are usually a function of spring degree day accumulation, following adequate winter chilling. These thermal cues will change as climates warm, and phenotypic plasticity between environments or over time often directly reflects changes in temperature regimes. Fall phenological traits including bud set, leaf abscission, and development of cold hardiness in perennials are usually triggered by photoperiod and therefore not likely to produce plastic responses as climates change; however, drought and other limiting environmental conditions can affect these responses. Differences in seasonal responses are well illustrated by recent studies of *Arabidopsis thaliana* (Springate et al. 2011). They imposed selection for earlier flowering in climatic conditions suitable for spring-annual and winter-annual life histories, in the ‘fast-cycler’ and ‘long-flowerer’ genotypes exhibiting these life history traits. Selection for early flowering in spring-annual conditions increased phenotypic plasticity, while selection for early flowering in winter-annual conditions reduced it.

The available length of favorable conditions for growth will lengthen in some environments for some populations due to a longer warm, frost-free period, but shorten for others due to increased drought. In an elevational reciprocal transplant study of two European deciduous tree species, *F. sylvatica* and *Quercus petraea*, both the timing of leaf emergence in spring, and the senescence of leaves at the end of the growing season showed greater variation due to phenotypic plasticity than genetic differences (Vitasse et al. 2010). Plasticity was high in both species, with leaf emergence advancing by an average of 5.7 days/°C increase, and slightly faster in oak than in beech, whereas genetic differences in leaf emergence date among populations were significant in beech but not in oak. While leaf emergence dates in spring advanced linearly with temperature, reaction norms for leaf senescence in fall were nonlinear with the greatest longevity of leaves in intermediate thermal environments. This was likely a result of moisture deficits resulting in premature senescence at high temperatures, and resulted in a nonlinear reaction norm between temperature and growing season length for all populations.

## Factors influencing evolutionary versus plastic responses

The studies compiled here demonstrate many plant populations can respond to climatic changes through plasticity or evolution. However, it is difficult to predict, for a particular population, whether plasticity or evolution is more important, and if these responses will be sufficient to prevent extinction. Being able to make such predictions is critical for conservation and management as climate change continues. While theoretical expectations can inform predictions of the capacity for rapid adaptation to climate change, little theory exists for phenotypic plasticity (Kopp and Hendry 2014), or for the interactions between adaptation and plasticity. While plasticity can be assessed experimentally in the ways discussed above, phenotypic buffering, producing stable phenotypes under a range of environmental conditions, may also be important (Reusch 2014). For a given population, a variety of factors influence the type and degree of climate change response. We briefly discuss several of these factors here. We also provide several predictions that can be tested in future work on responses to climate change.

### Climate change factors

Responses to climate change are likely to be highly dependent on the rate, scale, and predictability of environmental changes. The more rapid the environmental change, the faster the plastic or evolutionary response would need to be to prevent population extinction (Chevin and Lande 2010; Chevin et al. 2013). Because plastic changes can occur within a generation and evolutionary changes necessarily occur across generations, one prediction is that plasticity may be a more important immediate response to very rapid environmental changes. However, if the environmental changes are rapid enough that populations are experiencing novel conditions, evolutionary responses may be more important, especially over the long term. Ecological genetic theory suggests that responses are most likely to be plastic when the environment varies in a predictable way, and there is empirical support for this idea (Alpert and Simms 2002). While global temperatures are steadily increasing, there is also growing climatic instability, so climate change could lead to either increased or decreased plasticity.

### Capacity for plasticity and adaptation

Because the rate of adaptation is directly proportional to the degree of additive genetic variance in a population (Fisher 1958), factors influencing genetic variance are likely to be important for determining the ability of populations to adapt to climate change (Jump and Peñuelas 2005). Determining precisely which traits and underlying genes are most important under projected, dynamic climate scenarios can be empirically difficult due to trade-offs among suites of traits (Etterson and Shaw 2001; Aguilar et al. 2008). Initial responses to novel selection, however, are likely to be phenotypic (Bradshaw 1965), and the capacity for plasticity in important traits (e.g., bud break and growth initiation timing in spring; flowering time; drought tolerance; growth cessation and bud formation in late summer or fall) should play a major role in the early stages of population persistence (Jump and Peñuelas 2005). Studies that have considered the role of phenotypic plasticity in the context of climate change have found notable evidence that plasticity can aid in adaptation across a variety of species (Table 1). The occurrence and magnitude of phenotypic plasticity can vary genetically (Pigliucci 1996; DeWitt et al. 1998; de Jong and Gavrilets 2000; Schlichting and Smith 2002), and in some cases, plasticity may hinder evolutionary responses (Crispo et al. 2010). It is therefore necessary to consider factors influencing genetic composition within and among plant populations. Furthermore, life history and generation time may influence climate change responses, as plants with more generations in a given time period and plants with less delayed reproductive maturity may be able to adapt more quickly to changing conditions.

### Mating systems

Mating systems influence genetic variation within and among populations (Loveless and Hamrick 1984) and therefore may play an important role in the capacity for populations to adapt to novel environments. Plant populations can vary widely in mating system, from obligately outcrossing to completely self-fertilizing. Increased selfing has been associated with decreased additive genetic variation (Charlesworth and Charlesworth 1995; Bartkowska and Johnston 2009), and selfing populations may have smaller effective population sizes (Barrett and Kohn 1991; Charlesworth et al. 1993). In small populations, genetic drift can have a strong influence on fitness, by decreasing the efficacy of selection and increasing the chance of fixation of deleterious alleles or extinction (Lynch and Gabriel 1990; Lande 1994; Kopp and Hendry 2014). Outcrossing populations, therefore, may exhibit some genetic advantages over selfing populations via high within-population genetic variation, high gene flow and limited linkage disequilibrium (Glémin et al. 2006; Anderson et al. 2012a). For instance, one study found a strong effect of mating system on the level of genetic variance maintained or lost in fragmented habitats (Aguilar et al. 2008). For outcrossing to occur, however, many plants depend upon insect pollinators and those may suffer dramatic consequences from environment-induced changes in flowering phenology that may be asynchronous with pollinator activity, as well as general declines in occurrence and abundance of some species of insect pollinators (Parmesan 2006; Memmott et al. 2007; Potts et al. 2010; Forrest and Thomson 2011). Climate change may also change the dynamics and timing of wind, affecting mating success and gene flow levels and directions in outcrossing wind-pollinated species (Kremer et al. 2012).

The role of mating system in the ability of populations to adapt is likely to vary based on the type of pollination vector. In populations reliant on pollinators that are also facing strong selection via climate change or other pressures, more selfing may prove a superior strategy. On the other hand, highly fragmented populations with increased inbreeding may suffer consequences due to a lack of genetic variation. Interestingly, a review of the literature found no correlation between mating system and local adaptation (Hereford 2010). This emphasizes that although we can draw upon these studies to frame hypotheses, it is still unclear how mating system will influence the ability of plant populations to adapt to climate change.

### Gene flow

Based on the existing theoretical and empirical literature, we predict that gene flow will often increase the ability of populations to respond to climate change, although the introduction of maladaptive alleles could hinder adaptive responses. Gene flow in most plants occurs primarily through movement of pollen and also by movement of seeds. Gene flow can increase genetic variation and reduce inbreeding within populations, particularly as ranges of species shift (Petit et al. 2003; Savolainen et al. 2011; Kremer et al. 2012). Furthermore, admixture between genetically differentiated populations could facilitate adaptation to climate change via the introduction of locally adapted alleles (Jump and Peñuelas 2005; Yeaman and Jarvis 2006; Paul et al. 2011; Anderson et al. 2012a; Aitken and Whitlock 2013). For example, a study on two species of *Betula* found that gene flow from populations with earlier bud burst would likely be necessary for adaptation to climate change (Billington and Pelham 1991).

Gene flow among populations can also be maladaptive; for example, gene flow from large central populations to small peripheral ones is theorized to swamp local adaptation for marginal populations (Kirkpatrick and Barton 1997; Bridle and Vines 2007). The variable impacts of gene flow on fitness in a climate change context were shown in a recent study that used artificial crosses to mimic gene flow at the warming range limit in *Mimulus laciniatus* (Sexton et al. 2011). Artificial gene flow resulted in increased fitness at early life stages in the offspring of individuals from peripheral populations at the range margins, suggesting that inbreeding depression may limit adaptation and range expansion. In this example, gene flow from central to peripheral populations was maladaptive, while gene flow between edge populations increased fitness (Sexton et al. 2011).

In a changing climate, asymmetrical gene flow could facilitate adaptation at the leading edge of migration by providing a source of warmer-adapted alleles (Davis and Shaw 2001; Anderson et al. 2012a). For example, an experimental study of the plant *C. fasciculata* showed that gene flow from a southern Oklahoma population into a northern Minnesota population had an overall positive effect (Etterson 2008). A recent review of long-distance gene flow in trees found that the positive effects of gene flow, even across large spatial scales, likely outweigh maladaptive effects (Kremer et al. 2012). However, gene flow may disrupt adaptation to nonclimatic abiotic or biotic environmental factors such as soil type or microbes (Aitken and Whitlock 2013) and may have a deleterious effect on adaptation in rear-edge populations through swamping (Holliday et al. 2012). For populations that are isolated or fragmented, gene flow between neighboring populations could be rare and adaptation will necessarily depend on existing phenotypic variation and adaptive potential (Lande 1988).

## Future directions

The results of this review suggest that although interest in and research on plastic and evolutionary responses to climate change is growing, we still have relatively little empirical data for making species and trait-specific predictions about the likely rates and types of plant responses as climatic conditions continue to change. Not only are more field-based and experimental environment studies necessary with sufficiently large sample sizes to detect population changes over time (Kopp and Hendry 2014), but there is also an urgent need to create organized databases for synthetic analyses with multiple species, populations and locations. The establishment of data repositories such as Dryad is an important step in this direction, and the creation of standardized formats for data on plasticity and phenotypic evolution would be highly useful.

Another important future direction in the field is understanding the genetic basis of plastic and evolutionary responses to climate change (Franks and Hoffmann 2012). Understanding the genetic architecture of these responses may enhance our ability to predict the effects of climate change on natural populations. For example, we might expect that a trait controlled by a single gene of large effect and under strong selection by climate change factors is more likely to change in frequency and potentially sweep to fixation, leading to an adaptive change, than a trait controlled by a complex network of interacting genes. Studies on the genetic basis of adaptation to climate change can also provide more general insights into the molecular basis of plant responses to environmental variation. Current techniques in genomics and bioinformatics are making the technical aspects of this goal more feasible for nonmodel organisms (Reusch and Wood 2007; Rokas and Abbot 2009), providing several types of information that can be used in the assessment of the genetic basis of climate change responses. First, the genes underlying key climatic traits involved in adaptation to climate are being discovered through genome wide association studies (GWAS), candidate gene approaches, and allele-environment correlations. Second, polymorphisms in these genes (e.g., single-nucleotide polymorphisms) that are associated with phenotypes can be used to track allele frequency changes allochronically resulting from selection or synchronically through cohort studies in long-lived species. Third, phenotypic plasticity may be due in part to changes in gene expression, which are becoming easier to assess. Lastly, genomic tools to study epigenetic mechanisms such as DNA methylation are becoming more accessible for nonmodel organisms. However, as discussed above, we still lack material from good examples of evolutionary responses to climate change on which to conduct such studies. For plant studies, reciprocal transplant experiments with clonal material would be ideal for GWAS of climate-related traits, for tracking fitness in different environments over time, and for the study of relationships between epigenetic changes or changes in gene expression and phenotypic plasticity.

## Conclusions

We can draw several conclusions about phenotypic and evolutionary responses to climate change in plant populations. Although there are a modest number of studies to draw on, the number is growing quickly. Both plastic and evolutionary responses to climate change have been detected frequently, both can occur rapidly, and often both occur simultaneously. However, the extent of these responses may be overestimated, and the degree of response may not be sufficient to keep pace with current rates of climate change. There are relatively few studies that directly examine responses over time, that conclusively demonstrate adaptation or the causal climatic driver of the responses, or that quantitatively partition responses due to plasticity versus evolution. Additional studies designed to meet these objectives are needed to predict the response of plant populations to climate change. Despite these limitations, current evidence suggests that ongoing climate change is already having a substantial influence on the characteristics of terrestrial plant populations, and both plastic and evolutionary responses of these populations are being shaped by changes in climatic conditions. Just as investigating the historical occurrence and extent of local adaptation was a major scientific goal in the last and early part of this century, increasing our understanding of contemporary plastic and evolutionary responses to climate change is now a central objective of evolutionary and ecological genetics. Additional research in this area is likely to further illuminate our basic understanding of plasticity and evolution in natural populations, and increase our ability to protect and manage these populations as the climate continues to change.
